# Key Role of Reactive Oxygen Species (ROS) in Indirubin Derivative-Induced Cell Death in Cutaneous T-Cell Lymphoma Cells

**DOI:** 10.3390/ijms20051158

**Published:** 2019-03-07

**Authors:** Marwa Y. Soltan, Uly Sumarni, Chalid Assaf, Peter Langer, Ulrich Reidel, Jürgen Eberle

**Affiliations:** 1 Skin Cancer Centre Charité, Department of Dermatology and Allergy, Charité—Universitätsmedizin Berlin, Charitéplatz 1, 10117 Berlin, Germany; Marwayassin@med.asu.edu.eg (M.Y.S.); uly.sumarni@googlemail.com (U.S.); chalid.assaf@helios-gesundheit.de (C.A.); ulrich.reidel@charite.de (U.R.); 2 Department of Dermatology and Venereology, Faculty of Medicine, Ain Shams University, Cairo 11591, Egypt; 3 Clinic for Dermatology and Venereology, Helios Klinikum Krefeld, Lutherplatz 40, 47805 Krefeld, Germany; 4 Institute of Chemistry, University of Rostock, Albert-Einstein-Str. 3a, 18059 Rostock, Germany; peter.langer@uni-rostock.de; 5 Leibniz Institute of Catalysis at the University of Rostock e.V., Albert-Einstein-Str. 29a, 18059 Rostock, Germany

**Keywords:** CTCL, apoptosis, cell viability, c-FLIP, XIAP

## Abstract

Cutaneous T-cell lymphoma (CTCL) may develop a highly malignant phenotype in its late phase, and patients may profit from innovative therapies. The plant extract indirubin and its chemical derivatives represent new and promising antitumor strategies. This first report on the effects of an indirubin derivative in CTCL cells shows a strong decrease of cell proliferation and cell viability as well as an induction of apoptosis, suggesting indirubin derivatives for therapy of CTCL. As concerning the mode of activity, the indirubin derivative DKP-071 activated the extrinsic apoptosis cascade via caspase-8 and caspase-3 through downregulation of the caspase antagonistic proteins c-FLIP and XIAP. Importantly, a strong increase of reactive oxygen species (ROS) was observed as an immediate early effect in response to DKP-071 treatment. The use of antioxidative pre-treatment proved the decisive role of ROS, which turned out upstream of all other proapoptotic effects monitored. Thus, reactive oxygen species appear as a highly active proapoptotic pathway in CTCL, which may be promising for therapeutic intervention. This pathway can be efficiently activated by an indirubin derivative.

## 1. Introduction

Reactive oxygen species (ROS) play important roles in tissue damage and aging, as also addressed in this special issue. On the other hand, an increasing number of scientific studies in recent years indicated a particular role of ROS in apoptosis regulation in cancer cells. The mechanism(s) are still under discussion. Here, we give an example of cutaneous T-cell lymphoma (CTCL), where ROS is induced by the drug candidate indirubin.

Non-Hodgkin’s lymphomas (NHL) have shown increasing incidence in the last decades. About 5% of NHL are characterized by primary cutaneous manifestation through clonal proliferation of skin-homing memory T cells. This group of cutaneous T-cell lymphomas (CTCL) encloses Mycosis fungoides, Sézary syndrome and CD30(+) lymphoproliferative disorders. Cutaneous T-cell lymphomas represent a clinically and biologically distinct group of NHL without evidence for systemic disease at the time of first diagnosis. In clinical appearance and prognosis, they are clearly different from the histotypically cognate systemic lymphomas and their possible secondary cutaneous manifestations. Typically, they have the immunophenotype of CD3+ CD4+ CD45RO+ memory T-lymphocytes. While in its early stage CTCL may show an also indolent clinical course, it frequently transforms to a rapidly growing, malignant phenotype in later phases [1,2]. New treatments are needed particularly for these patients.

The elimination of tumor cells through the induction of apoptosis represents a principle goal in cancer treatment, and therapy resistance can thus be frequently explained by apoptosis deficiency [3]. Also, established therapies for CTCL as UV radiation or extracorporal photopheresis aim at an induction of apoptosis in tumor cells [4]. Extrinsic proapoptotic pathways are initiated by death ligands as CD95L/FasL or TRAIL (TNF-related apoptosis-inducing ligand), which also contribute to self-control of lymphocytes. Their binding to death receptors results in the formation of a death-inducing signaling complex, where initiator caspase-8 is activated [5]. Caspase-8 activation can be prevented by the competitive inhibitor protein c-FLIP (cellular FLICE-inhibitory protein) [6]. Initiator caspase-8 may cleave and activate the main effector caspase-3, which itself cleaves a large number of death substrates with the final result of DNA fragmentation [7]. Caspase-3 is negatively regulated through the binding of XIAP (chromosome X-linked inhibitor of apoptosis protein) [8].

In CTCL cells, the activation of the extrinsic caspase cascade plays a decisive role in controlling apoptosis. Thus, apoptosis resistance is correlated with reduced expression of the death receptor CD95/FAS [9] as well as with high and constitutive expression of c-FLIP [10]. Also, activation of the pro-survival transcription factors NF-κB [11] and STAT3 [12] were reported. In particular, different therapeutic strategies as NSAIDs, SAHA (suberoylanilide hydroxamic) and pentoxifylline resulted in downregulation of c-FLIP and XIAP in CTCL cells [13,14,15]. Finally, reactive oxygen species (ROS) may contribute to the regulation of apoptosis [16,17]. This is also suggested by enhanced levels of singlet oxygen (^1^O_2_) in the course of photodynamic therapy (PDT), used for the treatment of actinic keratosis [18,19] and also considered for CTCL [20]. However, the relation of ROS with described apoptosis pathways is still largely elusive.

The natural compound indirubin and a number of reported chemical derivatives are considered as candidates for cancer therapy. Indirubin was identified as an active component in a traditional Chinese medicine remedy (Danggui Longhui Wan), also applied for chronic myeloid leukemia. In clinical trials, indirubin has shown significant antitumor activity in chronic myeloid and chronic granulocytic leukemia [21,22]. Explaining the mode of action, a large number of intracellular targets have been described for indirubin derivatives, including cyclin-dependent kinases (CDK1, CDK2, CDK4 and CDK5), pRb, glycogen synthase kinase 3 (GSK-3), STAT3 (Signal transducer and activator of transcription), EGFR (Epidermal growth factor receptor), c-Jun, and JNK2 [23,24,25,26]. The activation of extrinsic apoptosis pathways by indirubin derivatives was found in melanoma cells [27,28]. To improve the anti-cancer activity of indirubin, we have previously introduced a series of chemical modifications [29,30,31]. Here, we investigated the direct effects of the indirubin derivative DKP-071 in CTCL cells. We furthermore unraveled its mode of action, which is based on caspase activation, downregulation of the caspase antagonists c-FLIP and XIAP and, in particular, on early production of ROS.

## 2. Results

### 2.1. Decreased Cell Proliferation and Viability Along with Induced Apoptosis by DKP-071

**Figure 1:**
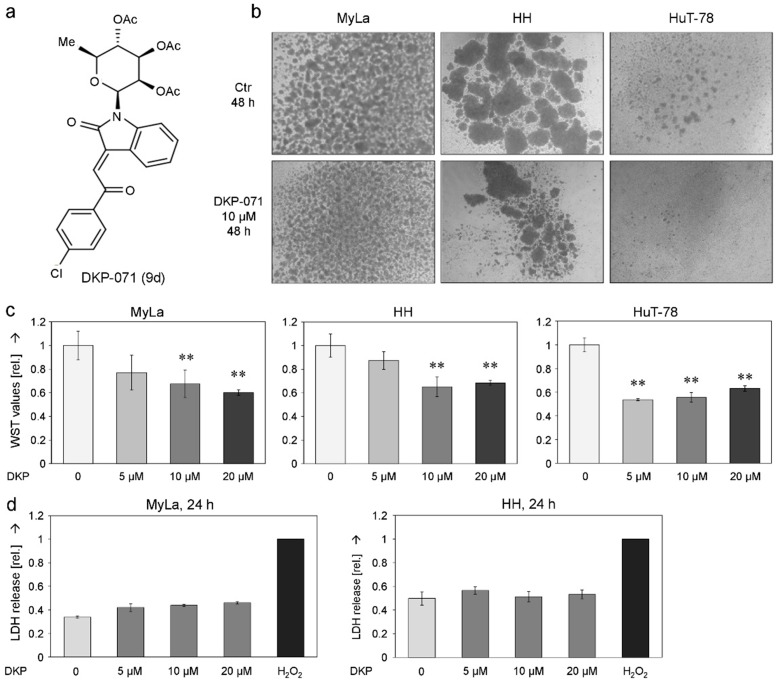
Decreased cell proliferation of CTCL cells by DKP-071. (**a**) Chemical structure of the indirubin derivative DKP-071 (termed substance 9d in [31]). (**b**) Cell cluster formation in CTCL cell lines MyLa, HuT-78 and HH. Control cells (Ctr) are shown vs. cells treated for 48 h with 10 µM DKP-071 (magnification: 1:40). Many independent experiments showed the same result. (**c**) Cell proliferation of MyLa, HuT-78 and HH, at 24 h in response to treatment with 5, 10 and 20 µM DKP-071 (DKP). Cell proliferation data were determined by WST-1 assay, and values are shown in relation (rel) to negative controls (0), which were set to “1”. Statistical significance is indicated (** *p* < 0.01). (**d**) Cytotoxicity was determined at 24 h in MyLa and in HH cells by LDH release assay. Values are shown in relation (rel) to H_2_O_2_-treated positive controls, which were set to “1”.

Synthesis and structural aspects of the indirubin derivative DKP-071/substance 9d (Figure 1a) have been reported previously [31]. Here, its effects in three CTCL cell lines MyLa, HuT-78 and HH were investigated. These cell lines are characterized by the formation of cell clusters, a typical lymphocyte differentiation step [32,33]. In a first approach, we observed reduced cell cluster size in response to DKP-071, likely indicating reduced T-cell activity (Figure 1b). In line with this, cell proliferation was significantly reduced at 24 h, as determined by WST-1 assay (Figure 1c). Reduced cell numbers were however not due to direct cytotoxicity, as lactate dehydrogenase (LDH) release assays at 24 h did not show a significant increase in MyLa or in HH cells (Figure 1d).

**Figure 2:**
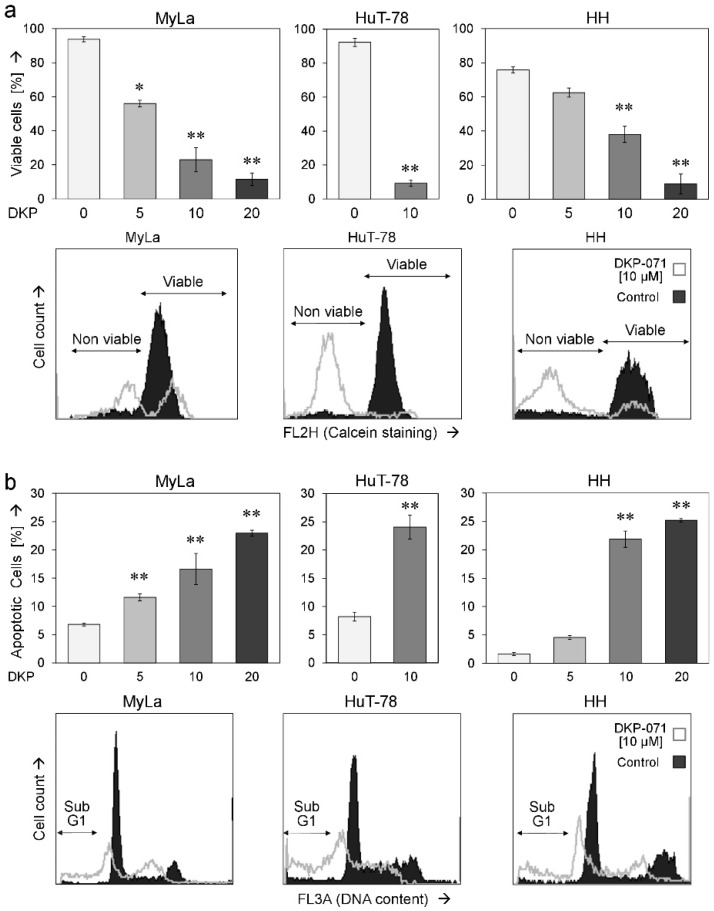
Reduced cell viability and induction of apoptosis. (**a**) Cell viability and (**b**) apoptosis were determined in three cell lines, in response to 48 h treatment with DKP-071 (5, 10 and 20 µM for MyLa and HH as well as 10 µM for HuT-78). Values were determined by calcein staining (**a**) and propidiumiodide staining (**b**), respectively. Characteristic histograms are shown for each cell line (10 µM treatment, overlays with controls); fractions of non-viable and viable as well as of apoptotic cells (sub-G1) are indicated. Mean values of triplicates +/− SDs of a representative experiment are shown. Statistical significance is indicated (treated cells vs. controls; * *p* < 0.05; ** *p* < 0.01).

Cell viability, as determined by calcein staining, was strongly decreased. A dose dependency (5–20 µM) was shown for MyLa and HH cells. At 48 h of treatment, 10 µM DKP-071 reduced the numbers of viable cells to 23% (MyLa), 9% (HuT-78) and 38% (HH), respectively (Figure 2a). Based on cell viability data, we calculated IC50 values of 7 µM DKP-071 for Myla and 11 µM for HH. For HuT-78, we used the WST data of Figure 1c, which resulted in an IC50 value of 8 µM for HuT-78. Loss of cell viability went along with an induction of apoptosis, which was determined by counting sub-G1 cells in cell cycle analyses. Induction of apoptosis showed a comparable dose dependency. At 48 h of treatment, 10 µM DKP-071 induced apoptosis in 17% (MyLa), 24% (HuT-78) and 22% of HH cells, respectively (Figure 2b). The concentration of 10 µM was selected for subsequent experiments.

### 2.2. Changes of Mitochondrial Membrane Potential and ROS Production

**Figure 3:**
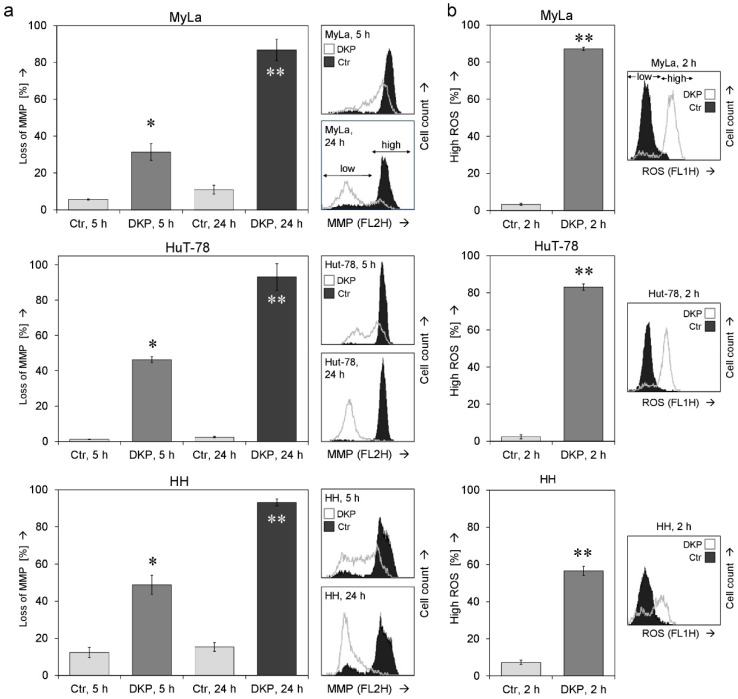
Effects on mitochondrial membrane potential and on ROS levels. (**a**) Relative changes in mitochondrial membrane potential (MMP) were determined at 5 h and 24 h in three CTCL cell lines in response to treatment with DKP-071 (10 µM). Mean values of triplicates +/− SD are shown; a second independent experiment series of MyLa revealed highly comparable results. Representative histograms (overlays of treated cells vs. controls) are given on the right side. (**b**) ROS levels were determined at 2 h of treatment. Mean values of triplicates +/− SD are shown; for MyLa, three independent experiments, each one with triplicates, revealed highly comparable results. Representative histograms (overlays of treated cells vs. controls) are given on the right side. Statistical significance is indicated (treated cells vs. controls; * *p* < 0.05; ** *p* < 0.01).

Questioning the mechanisms that mediate the antineoplastic effects of DKP-071 in CTCL cells, we determined the relative changes in the mitochondrial membrane potential (MMP) as well as relative levels of reactive oxygen species (ROS) in response to treatment. Loss of MMP, indicative for an activation of mitochondrial apoptosis pathways, already started in the three cell lines at 5 h (31–49%) but was much more evident at later time (24 h, 90% cells with low MMP; Figure 3a).

Reactive oxygen species (ROS) may mediate independent cell death pathways in cancer cells which are not yet completely understood [16]. Earlier than the loss of MMP, ROS levels were already strongly enhanced after 2 h. Thus, 87%, 83% and 57% of MyLa, HuT-78 and HH cells, respectively, showed high ROS levels at 2 h of DKP-071 treatment (Figure 3b).

### 2.3. Critical role of ROS for Proapoptotic Effects of DKP-071 

**Figure 4:**
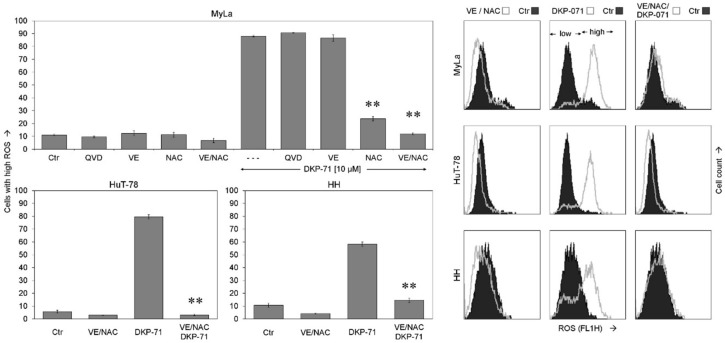
ROS suppression by antioxidative treatment. ROS levels are shown in MyLa in response to DKP-071 (10 µM). In addition, antagonists as vitamin E (VE, 1 mM), N-acetyl cysteine (NAC, 1 mM), the pancaspase inhibitor QVD-Oph (QVD, 10 µM), as well as combined NAC and VE (each 2 mM) were applied 1 h before DKP-071 treatment was started. Cells which received only DKP-071 but no antagonist are indicated by (- - -). The antioxidative effect was also shown in HuT-78 and in HH by the use of VE/NAC. Mean values of triplicates +/− SD of a representative experiment are shown; for MyLa, three independent experiments, each one with triplicates, revealed highly comparable results. Examples of flow cytometry measurement are shown on the right side as overlays versus control. Statistical significance of the differences of DKP-071/NAC-treated cells as well as DKP-071/VE/NAC-treated cells is indicated, each compared to the cells that received only DKP-071 (** *p* < 0.01).

To prove the significance of ROS as well as of caspase activation for the antineoplastic effects, the antioxidants tocopherol (vitamin E, VE) and N-acetyl cysteine (NAC) as well as the pan-caspase inhibitor QVD-Oph were applied. ROS production in response to DKP-071 was slightly reduced by VE and was strongly reduced by NAC, as shown in MyLa at 2 h. Most effective was a combination of VE and NAC (both at 2 mM, VE/NAC), which completely abolished ROS production after DKP-071 treatment in the three cell lines. QVD-Oph remained without effect on ROS, indicating that ROS was independent of caspase activity (Figure 4). 

**Figure 5:**
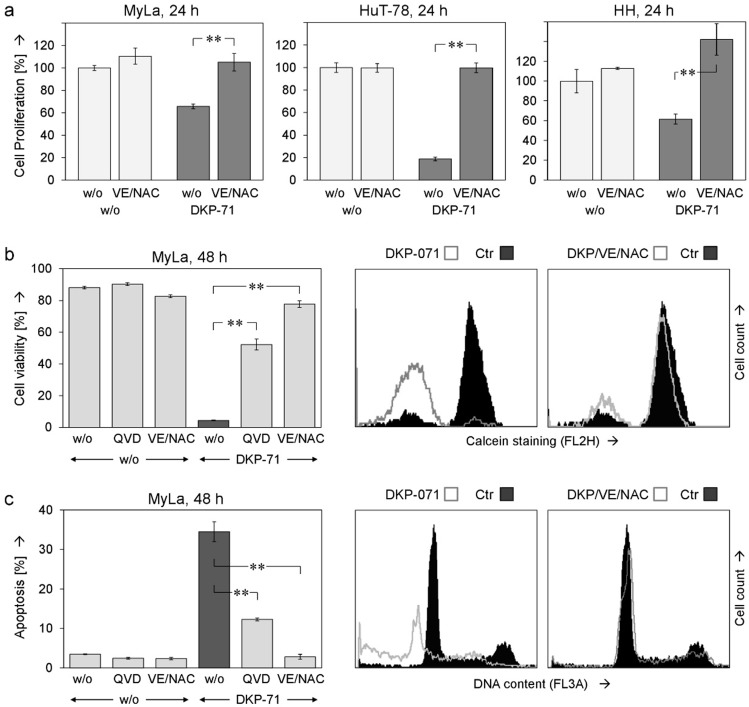
ROS suppression prevents apoptosis and restores cell viability. (**a**) Cell proliferation was determined at 24 h in response to DKP-071 as well as in response to the combination of NAC and VitE (2 mM; VE/NAC). Values were normalized to non-treated controls (100%). (**b**,**c**) Effects of agonists and antagonists on apoptosis induction (**b**) and cell viability (**c**), both at 48 h, are shown for MyLa cells. Examples of flow cytometry measurement are shown on the right side as overlays of treated cells versus controls. Mean values of triplicates +/− SD of representative experiments are shown; at least two independent experiments, each one with triplicates, revealed highly comparable results. Statistical significance of the differences between DKP-071/VE/NAC-treated cells to cells that received only DKP-071 is indicated (** *p* < 0.01).

ROS scavenging by VE/NAC proved the significant and upstream role of ROS for DKP-071-mediated effects. Thus, cell proliferation, which was decreased by DKP-071 at 24 h, was restored in three cell lines by VE/NAC (Figure 5a). Similarly, the effects of DKP-071 on cell viability were strongly diminished by VE/NAC as shown in MyLa at 48 h (from 4% to 78% viable cells, Figure 5b). Finally, the induction of apoptosis, induced by DKP-071 in MyLa at 48 h (35%), was completely prevented by VE/NAC (3%, Figure 5c). Caspase inhibition through QVD-Oph also diminished apoptosis and loss of cell viability, which was, however, less effective than the antioxydative treatment (Figure 4b,c). These findings support the explanation that ROS production was an upstream step.

### 2.4. Role of Caspases and Caspase Antagonistic Proteins

**Figure 6:**
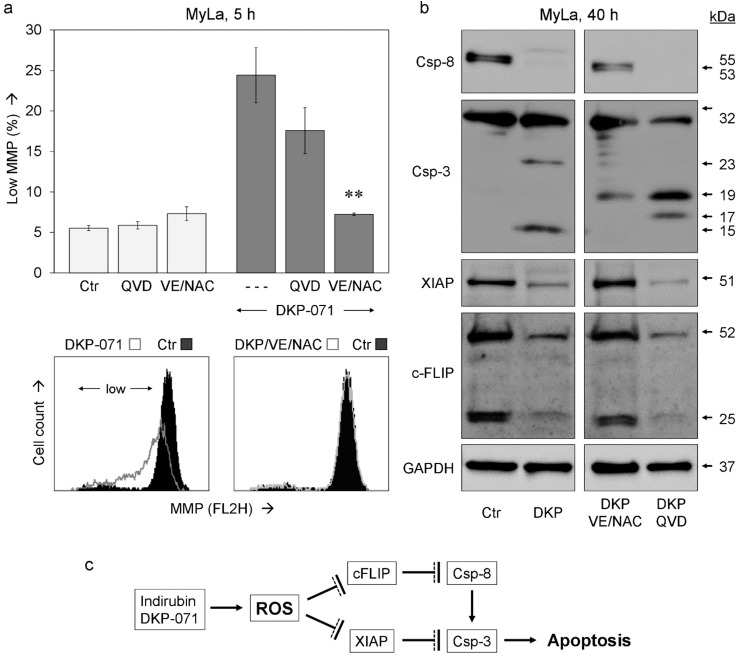
Effects on MMP and caspase cascade. (**a**) Effects of antioxidative treatment (VE/NAC, 2 mM) as well as of QVD-Oph (QVD, 10 µM) on mitochondrial membrane potential (MMP) are shown for MyLa cells at 5 h of DKP-071 treatment. Cells, which received only DKP-071 but no antagonist are indicated by (- - -). Mean values of triplicates +/− SD of a representative experiment are shown; two independent experiments, each one with triplicates, revealed highly comparable results. Statistical significance of the differences between DKP-071/VE/NAC-treated cells to cells that received only DKP-071 is indicated (** *p* < 0.01). Examples of flow cytometry measurement are shown below as overlays versus control. The cell population with low MMP is indicated. (**b**) Effects of DKP-071 and antioxidative pre-treatment on the expression of characteristic regulatory proteins of the extrinsic apoptosis caspase cascade were investigated by Western blotting. Each 30 µg of protein was loaded per lane, and blots were probed with antibodies for extrinsic initiator caspase-8 (proform, 53/55 kDa), main effector caspase-3 (proform, 32 kDa; cleavage products, 23, 19, 17, 15 kDa), caspase-3 antagonist XIAP (51 kDa) and caspase-8 antagonist c-FLIP (FLIP_L_, long, 52 kDa; FLIP_S_, short, 25 kDa). The housekeeping protein glyceraldehyde 3-phosphate dehydrogenase (GAPDH, 37 kDa) was used as loading control. Three independent series of protein extracts and Western blotting experiments revealed highly comparable results. (**c**) The pathway suggested for indirubin DKP-071-mediated apoptosis. Arrows indicate activation; blunt end lines indicate inhibition.

ROS also appeared upstream of MMP loss. Thus, VE/NAC almost completely prevented the loss of MMP in MyLa at 5 h (24% to 7%) whereas caspase inhibition was less effective here (18%, Figure 6a). Extrinsic caspase pathways are of major importance for apoptosis regulation in lymphoma cells, also including CTCL [10]. Thus, we investigated by Western blotting the activation/processing of initiator caspase-8 and the main effector caspase-3 as well as the expression of the caspase-3 antagonist XIAP and the caspase-8 antagonist c-FLIP.

In response to 40 h treatment with DKP-071, the proform of caspase-8 (53/55 kD) disappeared, indicating its complete processing. In parallel, Caspase-3 was processed to its mature, active cleavage product of 15 kDa. Importantly, VE/NAC strongly reduced the processing both of caspase-8 and caspase-3. In particular, no active cleavage product of caspase-3 (15 kDa) was detected, but the processing was stopped at an intermediate product of 19 kDa. In clear contrast, QVD-Oph remained without effect on caspase-8. It, however, prevented caspase-3 autoprocessing and halted the cascade at the 19 and 17 kDa intermediate cleavage products (Figure 6b, top). These findings clearly showed that ROS was upstream of any caspase regulation, while QVD-Oph acts downstream as a caspase-3 antagonist.

Explaining the activation of caspases, DKP-071 strongly reduced the expression of two most characteristic caspase antagonists, namely XIAP (51 kDa) and c-FLIP (long isoform, 52 kDa and short isoform, 25 kDa). Of particular note, this downregulation was completely prevented by antioxidants (VE/NAC), while caspase-3 inhibition through QVD-Oph remained without effect on c-FLIP and XIAP (Figure 6b, bottom). Thus, ROS was also upstream of the downregulation of c-FLIP and XIAP. These findings suggest a cascade in CTCL cells, leading from ROS production in response to DKP-071 treatment to c-FLIP and XIAP downregulation and further to caspase activation and apoptosis (Figure 6c).

## 3. Discussion

Many new therapies established in recent decades for most tumors have sometimes dramatically enhanced patients’ survival. Also for CTCL, present therapeutic options as topical steroids, bexarotene, phototherapy, interferon or some forms of targeted therapy have strongly improved the clinical outcome and often allow long-term survival. Nevertheless, in its late phase, CTCL may transform to a rapidly growing and ulcerating phenotype, characterized also by pronounced therapy resistance [1,2]. As apoptosis deficiency represents an in principal decisive issue in therapy resistance, the specific targeting of apoptosis pathways appears as a promising strategy [3].

Indirubin has been identified as the major component of a traditional Chinese medicine remedy, also applied for leukemia. In clinical trials for chronic myeloid and chronic granulocytic leukemia, it revealed significant antitumor activity, resulting in partial or complete remissions [21,22]. Chemical modifications of indirubin may further enhance its activity. Thus, we have recently reported the synthesis of new indirubin derivatives characterized by N-glycosylated 3-alkylideneoxindol structures [31]. This first report on the effects of an indirubin derivative in CTCL cells shows a particular high activity resulting in decreased cell proliferation and cell viability as well as induction of apoptosis. There is no cutaneous non-tumorigenic T-cell line, which could be used for investigations in vitro and prove the tumor-specificity of the effects. However, due to the only moderate side effects reported for indirubin in clinical trials [21,22], the here investigated indirubin derivative may be suggested as a potential new therapeutic option for CTCL. A proapoptotic strategy, as by DKP-071, may also apply for early CTCL, characterized by an indolent clinical course and apoptosis susceptibility.

As concerning the pathways, by which indirubins may exert their effects, multiple targets have been suggested including CDKs, GSK-3β, pRb, c-Src, FGF-R1, VEGFR, STAT3, c-Jun and JNK2 [23,24,25,26]. In melanoma cells, we have previously shown that indurubin derivatives enhance extrinsic apoptosis pathways as induced by TRAIL (TNF-related apoptosis-inducing ligand [27,28]. Extrinsic apoptosis pathways via caspase-8/caspase-3 are of particular importance for apoptosis regulation in CTCL cells [10,34]. Also in CTCL, DKP-071 mediated its proapoptotic effects via activation of the extrinsic caspase cascade, as shown by caspase-8 and caspase-3 processing as well as by the inhibition of the effects of indirubin through the caspase-3 antagonist QVD-Oph. Caspase activity is controlled by several antiapoptotic proteins such as the competitive caspase-8 antagonist c-FLIP and the protein family of cIAPs (cellular inhibitor of apoptosis proteins), e.g., the caspase-3 antagonist XIAP (chromosome X-linked) [6,8]. These two antagonists appeared as essentially involved in the present setting, as strongly downregulated by DKP-071 in CTCL cells. Downregulation of these two factors in CTCL has also been reported by other treatments. Thus, c-FLIP was downregulated in response to SAHA (suberoylanilide hydroxamic) and NSAIDs, while XIAP was downregulated in response to SAHA and pentoxifylline [13,14,15].

Reactive oxygen species (ROS) play important roles in tissue damage and aging, and antioxidative strategies were established to prevent these negative effects. Besides this, ROS may also be involved in proapoptotic signaling in cancer cells [16,28,35,36]. As an example from the clinic, photodynamic therapy (PDT), used for the treatment of actinic keratosis, results in the production of high amounts of singlet oxygen. However, the role of ROS in PDT is still controversially discussed [18,19,20]. ROS may act as a signaling molecule e.g., by affecting the mitochondrial membrane and thus activating intrinsic, mitochondrial apoptosis pathways, or it may provoke cellular damage e.g., by oxidizing membrane lipids resulting in necrosis or activation of a damage response.

Here, we show even another alternative. In CTCL, ROS acts in cellular signaling clearly upstream of the extrinsic apoptosis pathway. It is induced already at 2 h in response to indirubin treatment and resides upstream of the downregulation of XIAP and c-FLIP as well as upstream of the loss of MMP, suggesting a signaling cascade as shown in Figure 6c. Also for other treatments in CTCL cells, relations of ROS and apoptosis induction have been reported, such as for an inhibitor of the antioxidative protein mucin 1 [37], for silencing of the enhancer of zeste homolog 2 (EZH2) [38] and for doxycycline [39]. Thus, ROS production appears to be an important cellular signaling step related to the induction of apoptosis. The new proapoptotic pathways behind this may be useful for targeted cancer therapy. In CTCL, our data suggest a strong relation between ROS, caspase antagonists and the activation of the extrinsic apoptosis pathway. This signaling cascade is efficiently triggered by the indirubin derivative DKP-071, suggesting it as a therapeutic strategy for CTCL.

## 4. Materials and Methods

### 4.1. Cell Culture and Treatments

Three CTCL cell lines have been used here, which derive from patients with Mycosis fungoides and Sézary syndrome, respectively: Cell line MyLa was kindly supplied by Prof. K. Kaltoft, Århus University, Århus, DK. It derived from a plaque biopsy of a patient with MF [40]; HuT-78 was kindly supplied by Prof. R.C. Gallo, University of Maryland, Baltimore, MD, USA. [41] It derived from PBMCs of a patient with Sézary syndrome; and HH (CRL-2105, ATCC, Manassas, VA, USA) derived from peripheral blood of a patient with aggressive CTCL [42]. Cells were grown at 37 °C, 5% CO_2_ in RPMI 1640 medium supplemented with L-glutamine, 10% FCS and antibiotics (Biochrom, Berlin, Germany). Under these applied conditions, the cell lines revealed a similar growth behavior and proliferation rate; only the formation of cell clusters, typical for T-cells, varied considerably (Figure 1a).

For assays, cells were seeded in 24-well culture plates (100,000 cells and 500 µL per well) or in 96-well pates (40,000 cells and 200 µL per well). For protein extraction, cells were seeded in 6-well plates (400,000 per well, 2 mL). Treatments started at 24 h after seeding.

The indirubin derivative DKP-071 (termed substance 9d in [31]; Figure 1a) was used in concentrations of 5–20 µM. For caspase inhibition, cells were pre-incubated for 1 h with the pan-caspase inhibitor QVD-Oph (Sigma-Aldrich, Taufkirchen, Germany, 10 µM). For ROS scavenging, cells were pre-treated for 1 h with 1 mM α-tocopherol (vitamin E, Fluka, Steinheim, Germany), with N-acetylcysteine (NAC; Sigma-Aldrich, Taufkirchen, Germany; 1 mM) or with a combination of vitamin E and NAC (VE/NAC; each 2 mM).

### 4.2. Assays for Apoptosis, Cytotoxicity, Cell Viability and Cell Proliferation

Apoptotic cells were quantified as sub-G1 cells (less DNA than G1 cells) in cell cycle analyses. The assay reveals less DNA in apoptotic cells, because small DNA fragments are washed out from isolated nuclei [43]. Cells were harvested by centrifugation, lysed and stained for at least 1 h in hypotonic PI solution (40 µg/mL propidium iodide, Sigma-Aldrich, in 0.1% sodium citrate, 0.1% Triton X-100). Stained isolated nuclei were analyzed by flow cytometry at FL3A with a FACS Calibur (BD Bioscience, Bedford, MA, USA). Cells in G1, G2 and S-phase as well as sub-G1 cells were determined.

Cell cytotoxicity was determined by the analysis of cell supernatants for the activity of lactate dehydrogenase (LDH), which is released from cytotoxic cells in culture. Aliquots of cell supernatants were collected at 24 h, and LDH activity was quantified with a LDH activity assay (Cytotoxicity detection assay; Roche Diagnostics, Penzberg, Germany), following the protocol of the supplier. As positive controls, Triton X-100 (0.7%)-treated cells were used. Relative values were determined in an ELISA reader. The increased LDH activity in treated wells traces back to damaged, cytotoxic cells.

Cell viability was determined by staining cells with calcein-AM (PromoCell, Heidelberg, Germany), which is a cell-permeant non-fluorescent substance that is converted to green-fluorescent calcein in live cells by the activity of intracellular esterases. Cells, grown and treated in 24-well plates, were harvested after 24 h or 48 h by centrifugation. After washing 1× with PBS, they were resuspended in 200 µL of 2.5 µg/mL calcein-AM in PBS. Staining was done at 37 °C for 1 h. Labeled cells were washed again with 1 mL of PBS and were resuspended in 200 µL PBS. The percentage of viable cells was determined by flow cytometry (FL2H). In the calcein assay, the total cell number does not contribute to the results, only the percentage of active cells in the remaining cell population is determined.

Cell proliferation was determined by WST-1 assay (Roche Diagnostics). It depends on the cleavage of the water-soluble tetrazolium salt by mitochondrial dehydrogenases in metabolically active cells. CTCL cells were seeded in 96-well plates, and treatments were started after 24 h. At the time of analysis (24–48 h), WST-1 reagent was added for 1–2 h, and the absorbance at 450 nm was determined in an ELISA reader. Data were reported as the percentage of non-treated controls. The WST-1 assay gives an overview of all the antiproliferative effects of a given drug, which includes a lack of cells due to decreased cell proliferation as well as a lack of cells due to the induction of apoptosis or induction of cytotoxicity. Furthermore, the cell activity of the remaining cells contributes to the results.

### 4.3. Mitochondrial Membrane Potential (MMP) and Reactive Oxygen Species (ROS)

Changes to MMP over time or in response to drug treatment can be assessed by staining cells with the fluorescent dye TMRM^+^ (Tetramethylrhodamin-methylester, Sigma-Aldrich). Due to its positive charge, TMRM^+^ accumulates in negatively charged mitochondria. In the course of depolarisation of the mitochondria, (e.g., at the beginning of apoptosis), anions are released and TMRM^+^ concentrations decrease. This staining may also be used to determine the effectiveness of therapeutic compounds [44]. Cells grown and treated in 24-well plates were harvested and stained for 20 min at 37 °C with 1 µM TMRM^+^. After two-times washing with PBS, cells were analyzed by flow cytometry (FL2H).

Intracellular ROS levels were determined by the cell-permeable substance H_2_DCFDA (2′,7′-dichlorodihydrofluorescein diacetate; Thermo Fisher Scientific, Hennigsdorf, Germany). In cells with high ROS, the non-fluorescent H_2_DCFDA is oxidized and thus converted in the strongly fluorescent DCF (2′,7′-Dichlorfluorescein). Cells grown in 24-well plates were pre-treated for 1 h with H_2_DCFDA (10 µM), before starting treatment with effectors. After treatment, cells were harvested by centrifugation, washed with 1 mL PBS, resuspended in PBS, and analyzed by flow cytometry (FL1H). As positive controls, cells were treated with H_2_O_2_ (1 mM, 1 h).

### 4.4. Western Blotting

For Western blotting, total protein extracts were obtained by cell lysis in 150 mM NaCl, 1 mM EDTA, 2 mM PMSF, 1 mM leupeptin, 1 mM pepstatin, 0.5% SDS, 0.5% NP-40 and 10 mM Tris-HCl, pH 7.5. Western blotting on nitrocellulose membranes was performed as described previously [45]. Primary antibodies: Cleaved caspase-3 (9664, rabbit, 1:1000, Cell Signaling, Danvers, MA, USA); XIAP (#2042, rabbit, 1:1000, Cell Signaling); caspase-8 (#9746; mouse, 1:1000, Cell Signaling); c-FLIP (G-11, sc-5276; Santa Cruz, Heidelberg, Germany; 1:50); and GAPDH (sc-32233, mouse, 1:1000, Santa Cruz Biotech). Secondary antibodies: peroxidase-labelled goat anti-rabbit and goat anti-mouse (Dako, Hamburg, Germany; 1:5000).

### 4.5. Statistics

Assays described above were performed in triplicate determinations, and usually at least two completely independent series were performed for each experiment. For the determination of statistical significance, the values of all individual experiments were given together, after normalizing according to the controls. A Student’s *t*-test (two-tailed, heteroscedastic) was applied, and *p*-values of <0.05 were considered as statistically significant, while *p*-values of <0.01 were considered as highly significant. When applicable, significance is indicated in the bar charts (* for *p* < 0.05; ** for *p* < 0.01). The results of Western blots were reproduced in three independent series of experiments.

## 5. Conclusions

In summary, cutaneous T-cell lymphoma cells can be targeted by the induction of ROS. This results in an activation of the extrinsic apoptosis pathway via downregulation of c-FLIP and XIAP. This pathway is efficiently activated by an indirubin derivative, which thus represents an interesting candidate for therapy of cutaneous T-cell lymphoma.
